# Increased adenosine-to-inosine RNA editing in rheumatoid arthritis

**DOI:** 10.1016/j.jaut.2019.102329

**Published:** 2020-01

**Authors:** Nikolaos I. Vlachogiannis, Aikaterini Gatsiou, Domenico Alessandro Silvestris, Kimon Stamatelopoulos, Maria G. Tektonidou, Angela Gallo, Petros P. Sfikakis, Konstantinos Stellos

**Affiliations:** a First Department of Propaedeutic Internal Medicine and Joint Rheumatology Program, School of Medicine, National & Kapodistrian University of Athens, Athens, Greece; b Cardiovascular Disease Prevention Hub, Faculty of Medical Sciences, Newcastle University, Newcastle Upon Tyne, UK; c RNA Editing Lab, Oncohaematology Dept., Children Hospital Bambino Gesù IRCCS, Rome, Italy; d Department of Clinical Therapeutics, Alexandra Hospital, National & Kapodistrian University of Athens, Athens, Greece; e Freeman Hospital, Newcastle Upon Tyne Hospitals NHS Foundation Trust, Newcastle Upon Tyne, UK

**Keywords:** Rheumatoid arthritis, A-to-I RNA editing, *Alu* elements, Cathepsin S, EULAR responders, ADAR1

## Abstract

**Objective:**

Adenosine-to-inosine (A-to-I) RNA editing of *Alu* retroelements is a primate-specific mechanism mediated by adenosine deaminases acting on RNA (ADARs) that diversifies transcriptome by changing selected nucleotides in RNA molecules. We tested the hypothesis that A-to-I RNA editing is altered in rheumatoid arthritis (RA).

**Methods:**

Synovium expression analysis of ADAR1 was investigated in 152 RA patients and 50 controls. Peripheral blood mononuclear cells derived from 14 healthy subjects and 19 patients with active RA at baseline and after 12-week treatment were examined for ADAR1p150 and ADAR1p110 isoform expression by RT-qPCR. RNA editing activity was analysed by *AluSx*^*+*^ Sanger-sequencing of cathepsin S, an extracellular matrix degradation enzyme involved in antigen presentation.

**Results:**

ADAR1 was significantly over-expressed in RA synovium regardless of disease duration. Similarly, ADAR1p150 isoform expression was significantly increased in the blood of active RA patients. Individual nucleotide analysis revealed that A-to-I RNA editing rate was also significantly increased in RA patients. Both baseline ADAR1p150 expression and individual adenosine RNA editing rate of cathepsin S *AluSx*^*+*^ decreased after treatment only in those patients with good clinical response. Upregulation of the expression and/or activity of the RNA editing machinery were associated with a higher expression of edited *Alu*-enriched genes including cathepsin S and TNF receptor-associated factors 1,2,3 and 5.

**Conclusion:**

A previously unrecognized regulation and role of ADAR1p150-mediated A-to-I RNA editing in post-transcriptional control in RA underpins therapeutic response and fuels inflammatory gene expression, thus representing an interesting therapeutic target.

## Highlights

• The RNA editing enzyme ADAR1 is increased in rheumatoid arthritis.
• ADAR1-induced *Alu* A-to-I RNA editing is increased in active RA and decreases after treatment only in responding patients.
• Association of increased A-to-I RNA editing with pro-inflammatory gene expression suggests a role in chronic inflammation.
• Interrupting the pro-inflammatory ADAR1p150-induced *Alu* RNA editing may comprise an interesting therapeutic target in RA.

## 1 Introduction

Rheumatoid Arthritis (RA) is a chronic inflammatory rheumatic disease, which affects approximately 0.5–1% of the population worldwide [1]. To date RA remains an incurable disease, heavily affecting the quality of life of the patients, their ability to work, as well as their life expectancy overall [1]. Significant advances in the understanding of disease pathogenesis in the past decades, mainly the delineation of the pivotal role of tumor necrosis factor alpha (TNFα), have led to the development of biological therapies, which have transformed the course of these patients [2]. However, in 2019 remission and drug-free remission rates among RA patients remain unsatisfactorily low [1,3], with more than half of the patients treated with biological therapies showing persisting clinically active disease [4]. Moreover, there are currently no established biomarkers that can predict response to therapy [5]. Therefore, there is a demanding need to explore novel aspects underlying the pathogenetic mechanisms involved in RA.

Adenosine-to-inosine (A-to-I) RNA editing is the most abundant substitutional RNA modification, catalysed by the enzymes adenosine deaminases acting on RNA-1 and -2 (ADAR1 and ADAR2) [6]. A-to-I RNA editing takes place predominantly in repetitive non-coding regions of RNA, namely *Alu* elements*,* which have the ability to form double-stranded RNA regions, a pre-requisite for RNA editing [[7], [8], [9], [10], [11]]. *Alu* RNA editing is mainly, if not exclusively, catalysed by ADAR1 and is a widespread phenomenon, as *Alu* elements account for approximately 10% of the human genome [11]. Interestingly, presence of *Alu* elements in human genes has recently emerged as a critical regulator of RNA metabolism dictating the fate of the RNA molecules including alternative splicing, mRNA stability and translation [10,12]. More importantly, *Alu* elements are conserved only in primates, therefore *Alu* RNA editing constitutes an additional level of gene regulation specific for human disease [10,12,13]. Despite significant advances in the study of RNA editing with the introduction of second-generation RNA sequencing, the involvement of *Alu* RNA editing in human disease development and progression is currently poorly understood [12].

Herein, we tested the hypothesis that ADAR1 expression and A-to-I RNA editing are altered in the pro-inflammatory milieu of RA, probably influencing the expression of *Alu*-enriched pro-inflammatory genes. As an example, here, we focused on cathepsin S, a well-established target of ADAR1, whose mRNA stability and expression is controlled by *Alu* RNA editing [14]. Cathepsin S has an essential role in major histocompatibility complex (MHC)–II–mediated antigen presentation [15], autoantibody production [16] and extracellular matrix degradation [17], and has been previously reported to control experimental RA development in mouse [18], as well as to be increased in both the synovial fluid [19] and the systemic circulation of patients with RA [20].

## 2 Materials and methods

### 2.1 Analysis of RNA-sequencing dataset of synovial tissues

A large RNA-sequencing dataset (GSE89408) [21] including in total 202 synovial biopsy samples (normal joint = 28, osteoarthritis = 22, early RA-disease duration <1 year = 57, established RA = 95) was accessed through the NCBI database. Aligned data on ADAR1 and cathepsin S expression were extracted through the Gene Expression Omnibus database.

### 2.2 Analysis of RNA-sequencing dataset for ADAR1p110 and ADAR1p150 isoform expression

RNA-sequencing data (read length 101 bp generated from total RNA) from synovial samples of normal (28), osteoarthritis (22), early rheumatoid arthritis (57) and established rheumatoid arthritis (95) patients, were downloaded from the NCBI Sequence Read Archive (SRP092408) in .sra format and converted in .fastq by means of fastq-dump program that is part of the SRA toolkit package. Low-quality reads were discarded by filtering with the NGS QC Toolkit [22] and default parameters (cutoff read length for HQ = 70%, cutoff quality score = 20). High quality cleaned reads were mapped against pre-indexed human genome GRCh37, transcriptome (pre-processed set of known splice junctions from Ensembl annotation), and dbSNP common release 144 using HISAT2 version 2.1.0 [23] and default parameters. Unique and concordant alignments in .sam format were converted in the binary .bam format, sorted by genomic coordinates, and indexed by SAMtools. Transcriptome quantification was performed for each sample with StringTie v1.3.6 release [24] and differential expression was tested with the DESeq2 1.24.0 [25] (FDR ≤ 0.05) R package. Reference human transcriptome was obtained from UCSC (http://hgdownload.cse.ucsc.edu/goldenpath/hg19/database/). Expression of ADAR1 isoforms, ADAR1p150 (NM_001111) and ADAR1p110 (NM_001025107), was determined for each sample.

### 2.3 Patient recruitment and follow-up

Peripheral blood was collected in EDTA tubes (BD Vacutainer) from 19 consecutive consenting patients with RA fulfilling the 2010 ACR/EULAR criteria [26] and 14 apparently healthy controls (HC). All patients had active disease (DAS28-ESR: 3.4–7.1) at the time of sampling. Exclusion criteria included viral or bacterial infection during the past month and severe co-morbidities (cancer, heart or kidney failure). Clinical and laboratory RA features [28 tender/swollen joint count, erythrocyte sedimentation rate (ESR; mm/1st h.), C-reactive protein (CRP; mg/dl), visual analogue scale (VAS)-patient global] and rheumatoid factor (RF)/anti-cyclic citrullinated peptide (CCP) status were recorded at baseline **(**Table 1**)**.

**Table 1:** Demographics, clinical and laboratory features of controls and RA patients at baseline and after 12-week treatment.

	Rheumatoid Arthritis (n = 19)	EULAR Responders (n = 11)			EULAR moderate/non-responders (n = 8)		Controls (n = 14)
	Baseline	Baseline		12 weeks	Baseline	12 weeks	Baseline
Men/women, n	4/15	2/9			2/6		5/9
Age (mean ± SD, years)	54.2 ± 16.0	47.9 ± 14.8			62.8 ± 14.1		39.0 ± 12.6
Disease duration (mean ± SD, years)	7.3 ± 7.4	6.0 ± 7.2			9.0 ± 7.6		n/a
**Disease activity**							
Tender joint count (28), n	7.9 ± 5.3	7.3 ± 5.4		1.0 ± 1.8*	8.8 ± 5.5	5.4 ± 4.7*	0
Swollen joint count (28), n	5.4 ± 4.0	3.9 ± 3.8		0.8 ± 1.5*	7.5 ± 3.6	4.0 ± 3.2*	0
ESR (mm/1st h)	34 ± 23	33 ± 28		12 ± 15*	36 ± 15	26 ± 7*	n/a
CRP (mg/dl)	1.7 ± 1.5	1.6 ± 1.9		0.3 ± 0.3*	1.9 ± 0.9	0.9 ± 0.6*	n/a
DAS28-ESR	5.2 ± 1.2	5.0 ± 1.3		2.0 ± 0.8*	5.5 ± 1.1	4.5 ± 1.2*	n/a
**Auto-antibodies**							
RF positivity, n (%)	11 (57.9)	6 (54.5)			5 (62.5)		n/a
*anti*-CCP positivity, n (%)	12 (63.2)	7 (63.6)			5 (62.5)		n/a
**Therapy**							
csDMARDs, n (%)	5 (26.3)	3 (27.3)	6 (54.5)		2 (25.0)	4 (50.0)	0
corticosteroids, n (%)	6 (31.6)	3 (27.3)	5 (45.5)		3 (37.5)	4 (50.0)	0
*anti*-TNF, n (%)	0 (0.0)	0 (0.0)	6 (54.5)		0 (0.0)	5 (62.5)	0
*anti*-IL-1, n (%)	0 (0.0)	0 (0.0)	2 (18.2)		0 (0.0)	0 (0.0)	0
anti-CD20, n (%)	0 (0.0)	0 (0.0)	0 (0.0)		0 (0.0)	1 (12.5)	0

*ESR*: erythrocyte sedimentation rate; *CRP*: C-reactive protein; *DAS*: disease activity score; *RF*: rheumatoid factor; *anti-CCP*: anti-cyclic citrullinated peptide; *csDMARDS*: conventional synthetic disease-modifying antirheumatic drugs(methotrexate, leflunomide); *TNF*: tumor necrosis factor; *IL-1*: interleukin 1; *P < 0.05 compared to baseline.

RA patients were re-examined 12 weeks after initiation of new treatment (scale-up of treatment, conventional synthetic disease-modifying antirheumatic drugs (csDMARDS) ± corticosteroids and/or biological DMARDS) and were categorized in responders and moderate/non-responders according to EULAR's response criteria [27] (treatment modalities and their effect on disease characteristics in EULAR responders vs moderate/non-responders are shown in Table 1). For schematic representation of study design and patient recruitment see Supplementary Fig. 1. All participants gave written informed consent in compliance with the Declaration of Helsinki, which had been previously approved by the Ethics Committee of Laiko Hospital, Athens, Greece (Protocol Nr.:1368/17-11-2016), as well as by the Hellenic Data Protection Authority (Protocol Nr.:ΓΝ/ΕΞ/7901-2/22-12-2016).

### 2.4 Alu Sanger sequencing and RNA editing analysis

cDNA from peripheral blood mononuclear cells (PBMCs) was subjected to PCR with KOD Hot-start DNA polymerase (Millipore) for the amplification of the most edited *Alu* region in cathepsin S 3′ untranslated region (UTR), namely *AluSx*^*+*^ (PCR product size: 442bp) [14]. Gel-extracted PCR product was subjected to Sanger sequencing and editing rates of individual adenosines were determined following the analysis of the chromatopherograms as we have previously described [14].

### 2.5 Statistical analysis

Statistical analysis was conducted with SPSS 24.0. Normality of continuous variables was graphically assessed by histograms and P–P plots, as well as by Kolmogorov-Smirnov and Shapiro-Wilk tests. Pairwise differences were evaluated with two independent samples Student's t-test or the non-parametric Mann-Whitney *U* test for continuous variables between groups of RA and controls and chi-squared test for nominal variables. The Kruskal-Wallis test was used when comparing more than 2 groups. In order to account for multiple comparisons, Bonferroni correction was performed where applicable. Linear regression analysis was used to control for the effect of confounding factors. Correlations between continuous variables were explored with Pearson's test when normal distribution applied and Spearman's rank test for non-normal variables. Changes in continuous variables following treatment (same patients) were examined with the use of paired *t*-test or Wilcoxon signed-rank test for non-normal variables. Results were considered statistically significant when P < 0.05.

More details on PBMC isolation, RNA extraction, gene expression analysis, sample size calculation and patient and public involvement are given in **Supplementary methods**.

## 3 Results

### 3.1 The RNA editor ADAR1 is increased in RA synovium and peripheral blood

**Fig. 1:**
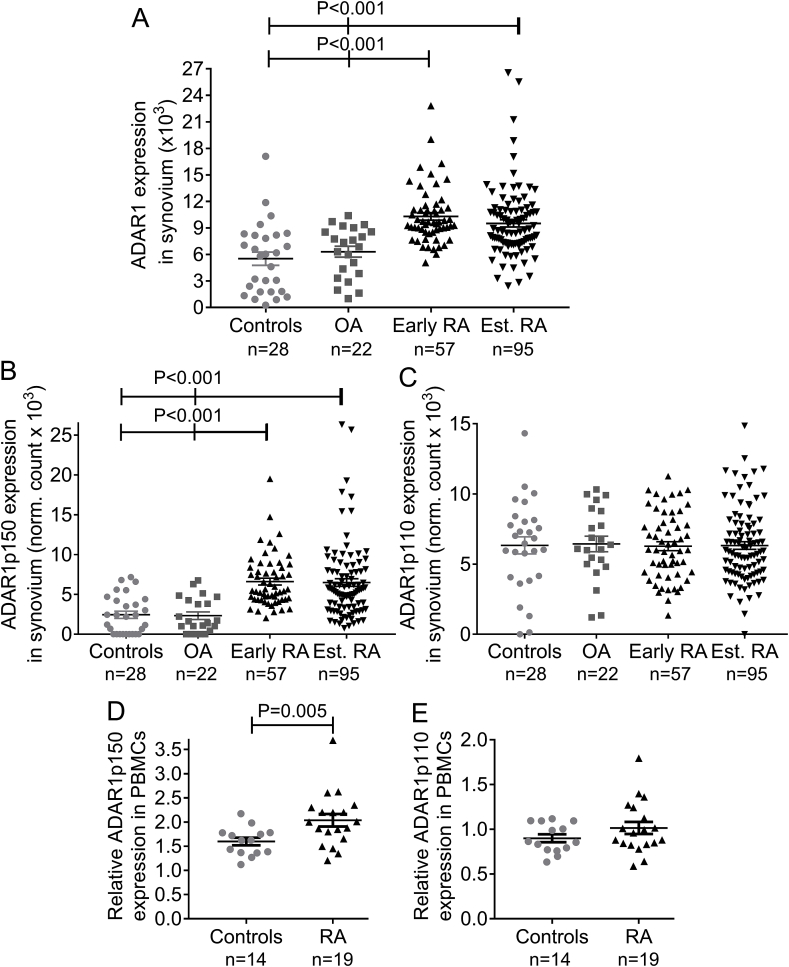
The RNA editing enzyme ADAR1 is increased in RA A,B,C. Expression analysis of ADAR1 mRNA levels and of its isoforms ADAR1p110 and ADAR1p150 at synovial biopsies derived from the RNA sequencing dataset GSE89408. The lines and P-values represent the comparison between early or established RA vs. normal synovium or osteoarthritis. **D,E.** mRNA expression levels of the interferon-inducible ADAR1p150 isoform (**D**) and the constitutively expressed ADAR1p110 isoform (**E**) in PBMCs of patients with active RA (n = 19) and controls (n = 14) as determined by qPCR with specifically designed Taqman primers (ADAR1p110: Hs01017596; ADAR1p150: Hs01020780, Applied Biosystems). Expression levels were normalized with the use of TBP as housekeeping gene (Hs00427621, Applied Biosystems). Lines in the scatter-plots represent mean ± SEM. P-values were produced by Mann-Whitney *U* test. *Est. RA*: established rheumatoid arthritis (>1 year disease duration); *OA*: osteoarthritis.

Expression analysis of the RNA-sequencing dataset GSE89408 revealed significant upregulation of total ADAR1 in synovium of patients with either early RA or established RA (P < 0.001 for each comparison: early or established RA vs. normal synovium or osteoarthritis, Fig. 1A). Interestingly there was a trend upregulation of ADAR1 in early RA when compared to established RA (P = 0.06 early vs. established RA). ADAR1 has two isoforms, namely ADAR1p110 and ADAR1p150 [6,28]. ADAR1p110 is constitutively expressed [6,28], while the interferon-inducible ADAR1p150 was reported to be also induced by the pro-inflammatory cytokines TNFα and interferon-γ [14]. Expression of ADAR1p150 isoform was significantly increased in rheumatoid synovium as well as in PBMCs of active RA patients (Fig. 1B,D), while mRNA levels of the constitutively expressed ADAR1p110 isoform did not differ between patients and controls (Fig. 1C,E). The expression levels of the second RNA editor ADAR2 (*ADARB1*) were similar in RA patients and healthy controls (Supplementary Fig. 2) indicating that ADAR1p150 is the only RNA editing enzyme induced under pro-inflammatory conditions in RA patients. Taken together these results suggest that total ADAR1 and particularly its pro-inflammatory isoform ADAR1p150 is increased in RA in both PBMCs and synovial tissue.

### 3.2 Increased Alu A-to-I RNA editing rate in active RA

**Fig. 2:**
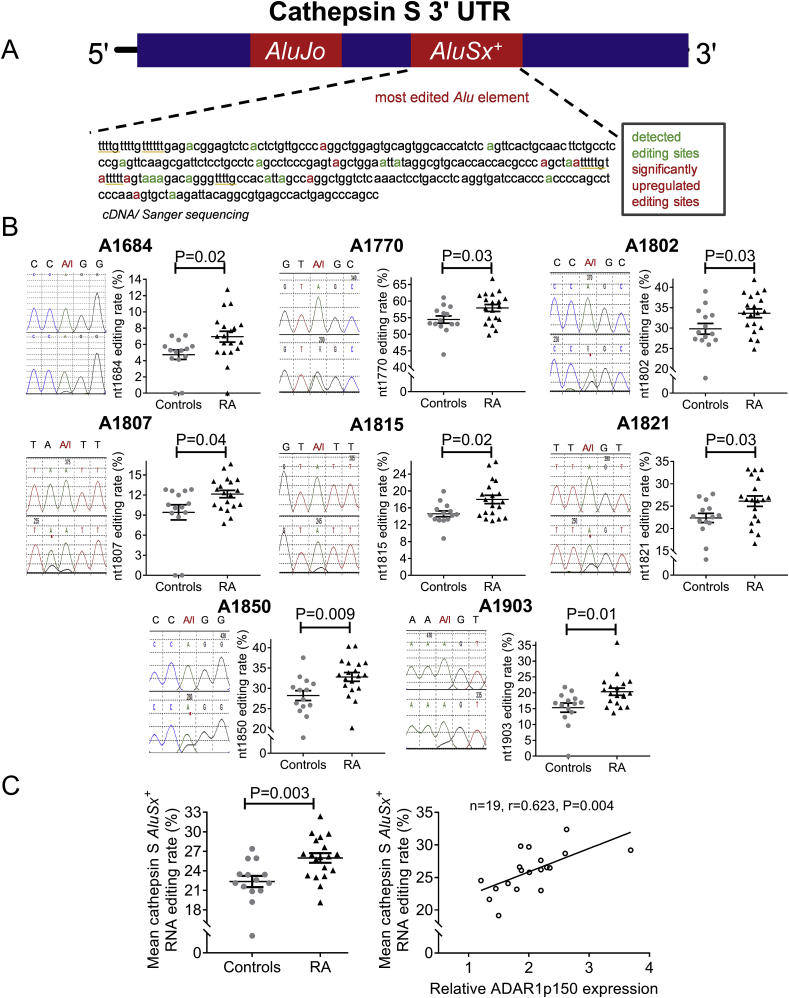
Increased *Alu* A-to-I RNA editing rate in active RA A. Schematic representation of cathepsin S 3′ UTR and cDNA sequence of cathepsin S *AluSx*^*+*^. All detected edited adenosines are depicted in green, while individual adenosines with significantly increased RNA editing rates in RA PBMCs are depicted in red. HuR binding sites are underlined. **B.** Chromatopherograms depict the genomic sequence (upper part) and the cDNA sequence (lower part) showing the RNA editing events (A-to-G mismatches). Scatter-plots depict RNA editing rate of 8 individual adenosines in PBMCs of active RA patients (n = 19) vs. healthy controls (n = 14). RNA editing rates of individual adenosines were determined following analysis of chromatopherograms. **C.** Scatter-plot (left) of mean cathepsin S *AluSx*^*+*^ RNA editing rate of the 8 edited adenosines in PBMCs from patients with active RA (n = 19) vs. controls (n = 14) and scatter-plot (right) showing correlation of individual RNA editing rates with ADAR1p150 mRNA levels in RA patients. Lines in the scatter-plots represent mean ± SEM. P-values were produced by Mann-Whitney *U* test/independent-samples *t*-test or Spearman's rank test, respectively. (For interpretation of the references to colour in this figure legend, the reader is referred to the Web version of this article.)

In order to evaluate the RNA editing activity of ADAR1, we sequenced the *AluSx*^*+*^ that is located in cathepsin S mRNA 3′ UTR (Fig. 2A), which is edited only by ADAR1 and is responsible for the post-transcriptional regulation of cathepsin S mRNA stability and expression [14]. Since inosines are recognized by the reverse transcriptase as guanosines, we could detect A-to-I RNA editing levels of modified adenosines as A-to-G nucleotide mismatches when we compare the RNA sequence with the genome. We were able to detect more than 20 edited adenosines (Supplementary Table 1). Both the average of all edited adenosines (P = 0.02) in *AluSx*^*+*^ as well as the individual RNA editing rate of 8 specific adenosines (Fig. 2B) were significantly higher in RA compared to controls (6–47% increase per adenosine, P < 0.05 for all, Fig. 2B). The RNA editing rates of 16 individual adenosines within cathepsin S 3′ UTR *AluSx*^*+*^ strongly correlated with ADAR1p150 mRNA levels (r = range 0.454–0.754, P ≤ 0.05 for all-Supplementary Table 1) The average RNA editing rate of the 8 significantly higher edited nucleotides (depicted in Fig. 2B) correlated with the expression of the pro-inflammatory inducible ADAR1p150 isoform (n = 19, r = 0.623, P = 0.004, Fig. 2C), but not with the constitutively expressed ADAR1p110 isoform (r = 0.265, P = 0.27).

### 3.3 Treatment of RA decreases A-to-I RNA editing levels only in responding patients

**Fig. 3:**
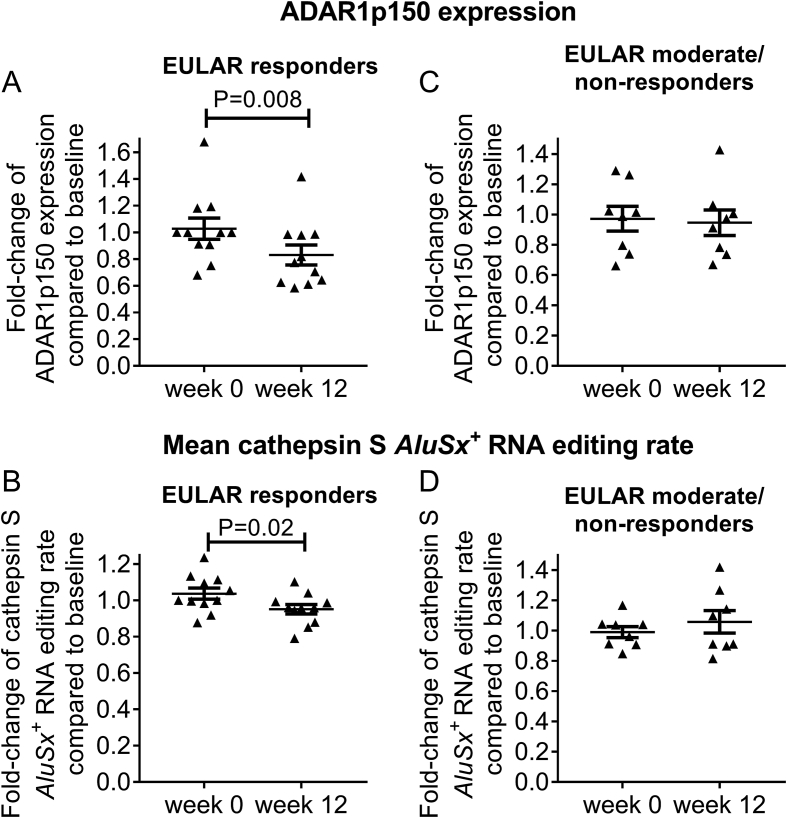
Effect of antirheumatic treatment on ADAR1p150 and RNA editing is dependent on clinical response**.** The effect of antirheumatic treatment on ADAR1p150 mRNA expression levels and cathepsin S *AluSx*^*+*^ RNA editing rate at baseline (week 0) and after 12-week treatment in EULAR responders (n = 11, **A,B**) and moderate/non-responders (n = 8, **C,D**). Individual ADAR1p150 expression levels and RNA editing rate of cathepsin S *AluSx*^*+*^ were standardized to the baseline median per group to show the effect of treatment (fold-change). Lines in the scatter-plots represent mean ± SEM. P-values were produced by Wilcoxon's signed-rank test or paired *t*-test.

Next, we investigated whether the observed upregulation of ADAR1p150 and *AluSx*^*+*^ RNA editing rate in active RA was reversible after 12 weeks of antirheumatic treatment with csDMARDS, corticosteroids and/or bDMARDs. Of note, both ADAR1p150 expression levels and average *AluSx*^*+*^ RNA editing rate decreased post-treatment only in EULAR responders (P = 0.008 and P = 0.02, respectively) (Fig. 3A and B), while they remained unaffected in those patients with moderate to no response (Fig. 3C and D). These results indicate that the effect of the antirheumatic treatment on RNA editing machinery depends on the clinical response to the given therapy, thus suggesting that the dynamic regulation of *Alu* A-to-I RNA editing reflects the course of RA.

### 3.4 Increased A-to-I RNA editing is associated with pro-inflammatory gene expression

**Fig. 4:**
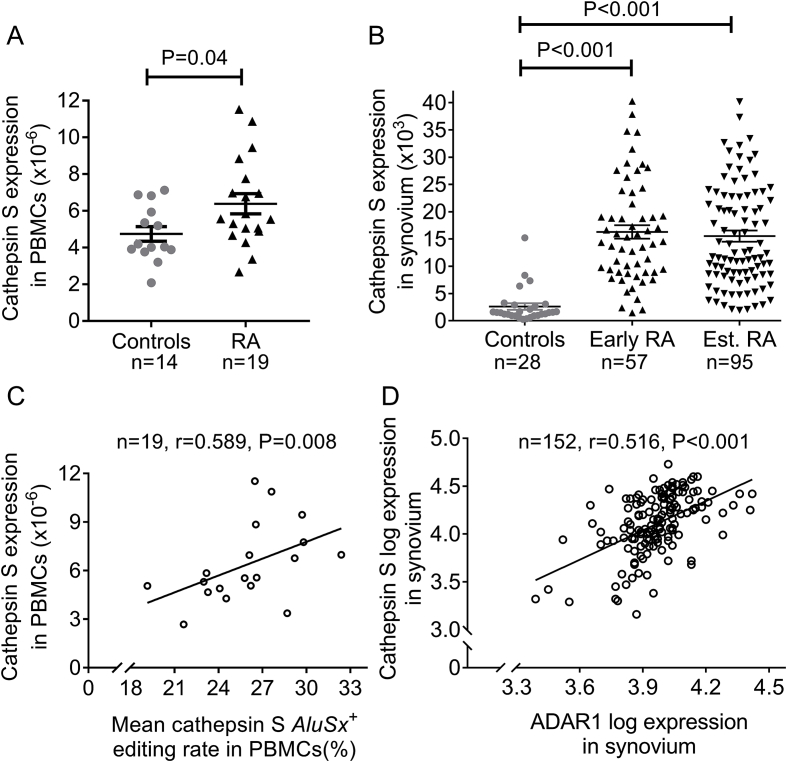
Cathepsin S mRNA expression in RA A. Cathepsin S mRNA expression in PBMCs of patients with active RA (n = 19) and controls (n = 14) quantified by RT-qPCR. **B.** Expression analysis of cathepsin S mRNA levels at synovial biopsies derived from the RNA sequencing dataset GSE89408. Aligned RNA-seq. data were downloaded from Gene Expression Omnibus. **C.** Scatterplot showing correlation of individual cathepsin S mRNA levels with RNA editing rate in active RA PBMCs. **D.** Scatterplot showing correlation of ADAR1 and cathepsin S mRNA levels at synovial tissue of RA patients from RNA-seq. dataset GSE89408; values are shown after logarithmic transformation. Lines in the scatter-plots represent mean ± SEM. P-values were produced by Mann-Whitney *U* test or Spearman's rank test/Pearson's test, respectively. *Est. RA*: established rheumatoid arthritis (>1 year disease duration).

A-to-I RNA editing of *Alu* elements disrupts the double-stranded RNA structure through the production of weak I–U bonds, unwinding double-stranded RNA secondary structure [10]. In this way, double-stranded RNA is regionally converted into a more single-stranded structure enabling the binding of single-strand RNA-binding proteins, such as HuR (*ELAVL1*), which can stabilize mRNAs and, thus, increase their expression, as we have previously described for cathepsin S mRNA [14]. Herein, we used cathepsin S, not only as a well-established target of ADAR1 [14] but also as an important molecule in RA development [18] to test whether the observed upregulation of ADAR1-mediated *Alu* RNA editing rate was associated with increased cathepsin S mRNA expression in RA. First, we confirm previous reports showcasing that cathepsin S mRNA expression was significantly increased in PBMCs of active RA patients (1.34-fold increase compared to healthy controls, P < 0.05, Fig. 4A), as well as at the synovium of RA patients (6-fold increase compared to normal synovium, P < 0.001, Fig. 4B). Most importantly, cathepsin S expression significantly correlated with the pro-inflammatory inducible ADAR1p150 isoform (r = 0.623, P = 0.004), as well as with the RNA editing rate of 12 individual adenosines within cathepsin S *AluSx*^*+*^ (r = range 0.461–0.716, P ≤ 0.05 for all; Supplementary Table 1) and mean RNA editing rate (r = 0.589, P = 0.008, Fig. 4C) in active RA. In contrast, no association was observed between the constitutively expressed ADAR1p110 isoform and cathepsin S mRNA levels (r = 0.268, p = 0.27). Next, we studied the association between the expression of ADAR1 and cathepsin S in a large cohort of RA synovial tissue. Interestingly, a similar correlation between ADAR1 and cathepsin S mRNA was also observed in synovial tissue derived from RA patients (n = 152, r = 0.516, P < 0.001, Fig. 4D) indicating that inflammation-induced *Alu* A-to-I RNA editing may augment cathepsin S mRNA.

Since RNA editing controls cathepsin S expression through increased HuR binding to *AluSx*^*+*^ [14], we statistically controlled for the potential involvement of HuR in regulation of cathepsin S expression by ADAR1p150-mediated RNA editing in RA. Towards this goal, we used a linear regression analysis for cathepsin S mRNA expression levels (dependent variable) and ADAR1p150 or average cathepsin S *AluSx*^*+*^ RNA editing rate controlling for the effect of HuR expression in these samples. Indeed, controlling for HuR abolished the observed relationship between cathepsin S mRNA expression and ADAR1p150 (P = 0.58) or average *AluSx*^*+*^ RNA editing (P = 0.29) suggesting that regulation of cathepsin S expression by RNA editing in RA is mainly mediated by HuR.

Of note, cathepsin S is only an example among several *Alu*-enriched pro-inflammatory genes, which are predicted to be highly edited according to the RNA editing database RADAR [29] and whose regulation by RNA editing in inflammatory disease warrants further investigation. For example, TNF receptor-associated factors TRAF1, TRAF2, TRAF3, TRAF5 have 10–117 *Alu* elements and 18-1135 predicted RNA editing sites, while when we analysed their expression in rheumatoid synovium, we found a significant correlation with ADAR1 expression (n = 152, r = range 0.356–0.743, Supplementary Table 2).

**Fig. 5:**
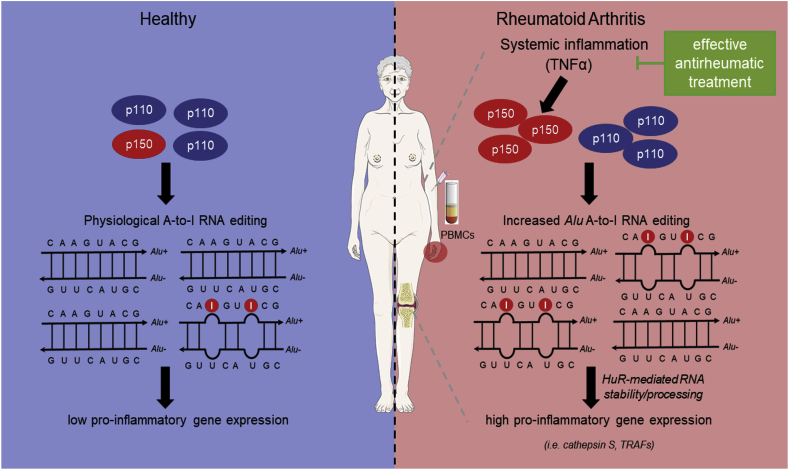
Proposed mechanism: Increased *Alu* A-to-I RNA editing fuels inflammatory gene expression in RA through post-transcriptional regulation of RNA metabolism. Synovial or systemic inflammation induces the expression of the pro-inflammatory inducible ADAR1p150 isoform and consequently A-to-I RNA editing of *Alu* elements. Increased *Alu* RNA editing may fuel inflammation by controlling the expression of inflammatory mediators through post-transcriptional HuR-dependent mRNA stability/processing. Certain items on this figure have been adapted from Servier Medical Art by Servier (https://smart.servier.com – licensed under Creative Commons Attribution 3.0 Unported License). *p110*: ADAR1p110, *p150*: ADAR1p150, *HuR*: human antigen R, *TRAFs*: TNF receptor-associated factors.

Taken together, our findings imply that *Alu* RNA editing may be a global primate-specific mechanism controlling *Alu*-enriched inflammatory mediators at post-transcriptional level through HuR-mediated RNA processing/stability in patients with RA. The proposed mechanism is summarized in Fig. 5.

## 4 Discussion

The present study provides first evidence that: 1) the RNA editor ADAR1, and specifically ADAR1p150 isoform, is increased in diseased synovium, the target tissue in RA, 2) the pro-inflammatory ADAR1p150 isoform as well as the A-to-I RNA editing rates of repetitive *Alu* elements are increased in circulating mononuclear cells from patients with active RA, 3) after a 12-week antirheumatic treatment reductions of ADAR1p150 expression and *Alu* A-to-I RNA editing rates are prominent only in patients with good clinical response and 4) increased *Alu* RNA editing rate is associated with increased pro-inflammatory gene expression in RA.

ADAR1-mediated RNA editing is indispensable for life, as mice lacking ADAR1 or having an editing-deficient knock-in mutation die *in utero* [[30], [31], [32]]. In humans, mutations in ADAR1 cause Aicardi-Goutières Syndrome associated with a type I interferon signature [33]. Further, ADAR1 and especially its isoform ADAR1p150 is a type I interferon-inducible gene [28]. Accordingly, ADAR1 expression was found to be increased in type I interferon-associated autoimmune diseases [[34], [35], [36], [37], [38], [39]], but also in other inflammatory diseases including acute myocardial infarction, atherosclerosis, cancer and viral infections [14,40,41]. More importantly, ADAR1 expression and activity are increased after stimulation with TNFα [14], the major cytokine that regulates the dynamics of transcriptome in RA [42], due to a significant increase in levels of ADAR1p150 isoform [14]. Our results may suggest that ADAR1 and specifically the ADAR1p150 isoform is increased in RA through a synergistic effect driven by TNF and type I interferon signaling. The upregulation of ADAR1 was found at the inflammatory tissues as well as in the circulation, and therefore, ADAR1 may have an impact on other autoimmune diseases besides RA. Further studies are warranted to elucidate the systematic and tissue- and cell-specific regulation and role of ADAR1, and more specifically of the pro-inflammatory ADAR1p150 isoform, in systemic autoimmunity.

ADAR1-induced A-to-I RNA editing takes place primarily in repetitive *Alu* elements, therefore comprising an upstream regulatory mechanism of RNA metabolism specific for primates [[8], [9], [10], [11], [12], [13]]. The widespread *Alu* elements have recently emerged as critical regulators of inflammation [43] and an enrichment of *Alu* elements has been recently reported in autoimmune diseases, potentially contributing to type I interferon pathway activation [43,44]. Data in systemic autoimmune disease are yet controversial, as studies have shown up-regulation of global *Alu* RNA editing index in peripheral blood of patients with systemic lupus erythematosus [38], but downregulation in keratinocytes of patients with psoriasis [39]. Clearly, comparative studies at transcriptome-wide level and especially at a single nucleotide or *Alu* level of individual transcripts in inflammatory and autoimmune diseases are warranted to enlighten the tissue-specific and disease-specific effects of *Alu* A-to-I RNA editing. The difference between global and transcript-specific RNA editing levels among the inflammatory and especially the autoimmune diseases may reflect not only the tissue-specificity of RNA editing [45], but also the inflammatory microenvironment and milieu in each disease [46]. We found that effective anti-inflammatory treatment was able to decrease RNA editing levels in our patients. To the best of our knowledge this is the first report showing the effect of anti-inflammatory treatment on RNA editing levels in association with clinical response. Whether RNA editing is a predictive biomarker of clinical response and/or involved in the response to therapy remains to be investigated in future studies.

*Alu* RNA editing can control various aspects of RNA metabolism including splicing and mRNA stability, which can ultimately affect expression levels of edited genes [10,12,47,48]. One of the major mechanisms leading to increased mRNA stability is the binding of the stabilizing RNA-binding protein HuR to its target motifs TTTTG, TTTTT and ATTTA located within the *Alu* elements [47,49]. Therefore, we also checked whether increased RNA editing observed in active RA patients led to aberrant gene expression, serving probably as an additional, primate-specific regulatory mechanism at the post-transcriptional level. Towards this goal, we used as exemplar the well-established ADAR1-target cathepsin S [14]. Cathepsin S belongs to the family of lysosomal cysteine proteases, which are critically involved in antigen presentation and immune response [50]. In mice, knockout of cathepsin S prevents collagen-induced arthritis [18], whereas increased circulating cathepsin S protein levels have also been detected in RA patients [20]. Moreover, cathepsin S may contribute to autoantibody production since it specifically mediates degradation of the invariant chain Ii, thereby promoting MHC-II antigen binding [15,16]. Previous studies have shown a significant upregulation of cathepsin S in RA synovial fluid [19], suggesting a role in synovial inflammation by its elastolytic properties and/or by enhancing antigen presentation and autoantibody production.

Our findings show a significant increase of cathepsin S both at the synovium and PBMCs of patients with RA which is significantly associated with the expression of ADAR1 and especially of ADAR1p150, as well as with the individual RNA editing rate of the adenosines located within the *AluSx*^*+*^ of cathepsin S 3′ UTR. These associations imply that the disruption of *AluSx*^*+*^ double-stranded RNA structure to a more single-stranded one by A-to-I RNA editing reveals the HuR target-sequences enabling the single-strand RNA-binding protein HuR to bind to its target cathepsin S, thus increasing its mRNA stability and expression, as previously evidenced in a series of mechanistic studies [14]. Of interest, cathepsin S is only one of the many edited *Alu*-enriched genes, e.g. TRAFs, whose expression was found to be associated with ADAR1 in a large cohort of RA patients, suggesting that our proposed mechanism (Fig. 5) may be applicable to a large number of pro-inflammatory genes in RA or other inflammatory diseases. Further studies are warranted to evaluate the role of A-to-I RNA editing in transcriptome metabolism and cellular function in RA.

## 5 Conclusions

To conclude, our data reveal a previously unrecognized dynamic regulation of *Alu* A-to-I RNA editing in RA that underpins therapeutic response and fuels inflammatory gene expression. The pro-inflammatory inducible ADAR1p150 isoform acts as a transcriptome-wide regulator of human-specific *Alu*-enriched inflammatory gene expression and, thus, may comprise an interesting predictive biomarker and a potential therapeutic target in patients with chronic inflammatory (auto)immune-mediated diseases, as supported by recent promising results in preclinical cancer models [51,52].

## Declarations of interest

None.

## Conflicts of interest

There are no competing interests for any author.

## Contributorship

K.Stellos and P.P.S. designed and guided research; N.I.V., M.G.T. and P.P.S. recruited the PBMC cohort; N.I.V. and A.Gatsiou performed research; D.A.S. and A.Gallo performed the analysis of RNA-seq. data for ADAR1 isoform expression; N.I.V., K.Stamatelopoulos, P.P.S. and K.Stellos analyzed data; A.Gatsiou, K.Stamatelopoulos, M.G.T., P.P.S and K.Stellos gave conceptual advice; and N.I.V. drafted the paper with input from all authors.

## Funding info

This work was supported by the 10.13039/100010663European Research Council (ERC) [Grant name: “MODVASC”, number: 759248] and the 10.13039/100004807DFG (SFB834 project number 75732319) to K. Stellos; ELKE grants from the 10.13039/501100005187National and Kapodistrian University of Athens, Greece to P.P. Sfikakis [grant number: 0974]; AIRC IG n. 22080 to A.Gallo; and a scholarship for doctoral studies to N.I.V by A. Onassis Public Benefit Foundation.

## Ethical approval information

All participants gave written informed consent, which had been previously approved by the Ethics Committee of Laiko Hospital, Athens, Greece (Protocol Nr.:1368/17-11-2016), as well as by the Hellenic Data Protection Authority (Protocol Nr.:ΓΝ/ΕΞ/7901-2/22-12-2016).

## Data sharing statement

RNA-sequencing dataset GSE89408 is publicly available at GEO database (NCBI). Raw data of the qPCR experiments and RNA editing analysis are available by the corresponding author upon reasonable request.

## Patient and public involvement

We did not involve patients or the public in our work.

## References

[1] Aletaha D., Smolen J.S. (2018). Diagnosis and management of rheumatoid arthritis: a review. JAMA.

[2] Sfikakis P.P. (2010). The first decade of biologic TNF antagonists in clinical practice: lessons learned, unresolved issues and future directions. Curr. Dir. Autoimmun..

[3] Aga A.-B., Lie E., Uhlig T., Olsen I.C., Wierød A., Kalstad S., Rødevand E., Mikkelsen K., Kvien T.K., Haavardsholm E.A. (2015). Time trends in disease activity, response and remission rates in rheumatoid arthritis during the past decade: results from the NOR-DMARD study 2000-2010. Ann. Rheum. Dis..

[4] Ljung L., Rantapää-Dahlqvist S., Jacobsson L.T.H., Askling J. (2016). Response to biological treatment and subsequent risk of coronary events in rheumatoid arthritis. Ann. Rheum. Dis..

[5] Tweehuysen L., van den Ende C.H., Beeren F.M.M., Been E.M.J., van den Hoogen F.H.J., den Broeder A.A. (2017). Little evidence for usefulness of biomarkers for predicting successful dose reduction or discontinuation of a biologic agent in rheumatoid arthritis: a systematic review. Arthritis Rheum..

[6] Nishikura K. (2010). Functions and regulation of RNA editing by ADAR deaminases. Annu. Rev. Biochem..

[7] Ramaswami G., Zhang R., Piskol R., Keegan L.P., Deng P., O'Connell M.A., Li J.B. (2013). Identifying RNA editing sites using RNA sequencing data alone. Nat. Methods.

[8] Athanasiadis A., Rich A., Maas S. (2004). Widespread A-to-I RNA editing of Alu-containing mRNAs in the human transcriptome. PLoS Biol..

[9] Bazak L., Haviv A., Barak M., Jacob-Hirsch J., Deng P., Zhang R., Isaacs F.J., Rechavi G., Li J.B., Eisenberg E., Levanon E.Y. (2014). A-to-I RNA editing occurs at over a hundred million genomic sites, located in a majority of human genes. Genome Res..

[10] Nishikura K. (2016). A-to-I editing of coding and non-coding RNAs by ADARs. Nat. Rev. Mol. Cell Biol..

[11] Kim D.D.Y. (2004). Widespread RNA editing of embedded Alu elements in the human transcriptome. Genome Res..

[12] Gatsiou A., Vlachogiannis N., Lunella F.F., Sachse M., Stellos K. (2018). Adenosine-to-Inosine RNA editing in health and disease. Antioxid. Redox Signal.

[13] Gatsiou A., Stellos K. (2018). Dawn of epitranscriptomic medicine. Circ. Genomic Precis. Med..

[14] Stellos K., Gatsiou A., Stamatelopoulos K., Perisic Matic L., John D., Lunella F.F., Jaé N., Rossbach O., Amrhein C., Sigala F., Boon R.A., Fürtig B., Manavski Y., You X., Uchida S., Keller T., Boeckel J.-N., Franco-Cereceda A., Maegdefessel L., Chen W., Schwalbe H., Bindereif A., Eriksson P., Hedin U., Zeiher A.M., Dimmeler S. (2016). Adenosine-to-inosine RNA editing controls cathepsin S expression in atherosclerosis by enabling HuR-mediated post-transcriptional regulation. Nat. Med..

[15] Riese R.J., Wolf P.R., Brömme D., Natkin L.R., Villadangos J.A., Ploegh H.L., Chapman H.A. (1996). Essential role for cathepsin S in MHC class II–associated invariant chain processing and peptide loading. Immunity.

[16] Saegusa K., Ishimaru N., Yanagi K., Arakaki R., Ogawa K., Saito I., Katunuma N., Hayashi Y. (2002). Cathepsin S inhibitor prevents autoantigen presentation and autoimmunity. J. Clin. Invest..

[17] Fonović M., Turk B. (2014). Cysteine cathepsins and extracellular matrix degradation. Biochim. Biophys. Acta.

[18] Nakagawa T.Y., Brissette W.H., Lira P.D., Griffiths R.J., Petrushova N., Stock J., McNeish J.D., Eastman S.E., Howard E.D., Clarke S.R. (1999). Impaired invariant chain degradation and antigen presentation and diminished collagen-induced arthritis in cathepsin S null mice. Immunity.

[19] Pozgan U., Caglic D., Rozman B., Nagase H., Turk V., Turk B. (2010). Expression and activity profiling of selected cysteine cathepsins and matrix metalloproteinases in synovial fluids from patients with rheumatoid arthritis and osteoarthritis. Biol. Chem..

[20] Ruge T., Södergren A., Wållberg-Jonsson S., Larsson A., Ärnlöv J. (2014). Circulating plasma levels of cathepsin S and L are not associated with disease severity in patients with rheumatoid arthritis. Scand. J. Rheumatol..

[21] Guo Y., Walsh A.M., Fearon U., Smith M.D., Wechalekar M.D., Yin X., Cole S., Orr C., McGarry T., Canavan M., Kelly S., Lin T.-A., Liu X., Proudman S.M., Veale D.J., Pitzalis C., Nagpal S. (2017). CD40L-Dependent pathway is active at various stages of rheumatoid arthritis disease progression. J. Immunol..

[22] Patel R.K., Jain M. (2012). NGS QC Toolkit: a toolkit for quality control of next generation sequencing data. PLoS One.

[23] Kim D., Langmead B., Salzberg S.L. (2015). HISAT: a fast spliced aligner with low memory requirements. Nat. Methods.

[24] Pertea M., Pertea G.M., Antonescu C.M., Chang T.-C., Mendell J.T., Salzberg S.L. (2015). StringTie enables improved reconstruction of a transcriptome from RNA-seq reads. Nat. Biotechnol..

[25] Love M.I., Huber W., Anders S. (2014). Moderated estimation of fold change and dispersion for RNA-seq data with DESeq2. Genome Biol..

[26] Aletaha D., Neogi T., Silman A.J., Funovits J., Felson D.T., Bingham C.O., Birnbaum N.S., Burmester G.R., Bykerk V.P., Cohen M.D., Combe B., Costenbader K.H., Dougados M., Emery P., Ferraccioli G., Hazes J.M.W., Hobbs K., Huizinga T.W.J., Kavanaugh A., Kay J., Kvien T.K., Laing T., Mease P., Ménard H.A., Moreland L.W., Naden R.L., Pincus T., Smolen J.S., Stanislawska-Biernat E., Symmons D., Tak P.P., Upchurch K.S., Vencovský J., Wolfe F., Hawker G. (2010). 2010 Rheumatoid arthritis classification criteria: an American College of Rheumatology/European League against Rheumatism collaborative initiative. Arthritis Rheum..

[27] Fransen J., van Riel P.L.C.M. (2005). The disease activity score and the EULAR response criteria. Clin. Exp. Rheumatol..

[28] George C.X., Samuel C.E. (1999). Human RNA-specific adenosine deaminase ADAR1 transcripts possess alternative exon 1 structures that initiate from different promoters, one constitutively active and the other interferon inducible. Proc. Natl. Acad. Sci..

[29] Ramaswami G., Li J.B. (2014). RADAR: a rigorously annotated database of A-to-I RNA editing. Nucleic Acids Res..

[30] Pestal K., Funk C.C., Snyder J.M., Price N.D., Treuting P.M., Stetson D.B. (2015). Isoforms of RNA-editing enzyme ADAR1 independently control nucleic acid sensor MDA5-driven autoimmunity and multi-organ development. Immunity.

[31] Liddicoat B.J., Piskol R., Chalk A.M., Ramaswami G., Higuchi M., Hartner J.C., Li J.B., Seeburg P.H., Walkley C.R. (2015). RNA editing by ADAR1 prevents MDA5 sensing of endogenous dsRNA as nonself. Science.

[32] Mannion N.M., Greenwood S.M., Young R., Cox S., Brindle J., Read D., Nellåker C., Vesely C., Ponting C.P., McLaughlin P.J., Jantsch M.F., Dorin J., Adams I.R., Scadden A.D.J., Ohman M., Keegan L.P., O'Connell M.A. (2014). The RNA-editing enzyme ADAR1 controls innate immune responses to RNA. Cell Rep..

[33] Rice G.I., Kasher P.R., Forte G.M.A., Mannion N.M., Greenwood S.M., Szynkiewicz M., Dickerson J.E., Bhaskar S.S., Zampini M., Briggs T.A., Jenkinson E.M., Bacino C.A., Battini R., Bertini E., Brogan P.A., Brueton L.A., Carpanelli M., De Laet C., de Lonlay P., del Toro M., Desguerre I., Fazzi E., Garcia-Cazorla À., Heiberg A., Kawaguchi M., Kumar R., Lin J.-P.S.-M., Lourenco C.M., Male A.M., Marques W., Mignot C., Olivieri I., Orcesi S., Prabhakar P., Rasmussen M., Robinson R.A., Rozenberg F., Schmidt J.L., Steindl K., Tan T.Y., van der Merwe W.G., Vanderver A., Vassallo G., Wakeling E.L., Wassmer E., Whittaker E., Livingston J.H., Lebon P., Suzuki T., McLaughlin P.J., Keegan L.P., O'Connell M.A., Lovell S.C., Crow Y.J. (2012). Mutations in ADAR1 cause Aicardi-Goutières syndrome associated with a type I interferon signature. Nat. Genet..

[34] Crow M.K., Wohlgemuth J. (2003). Microarray analysis of gene expression in lupus. Arthritis Res. Ther..

[35] Laxminarayana D., Khan I.U., Kammer G.M. (2002). Transcript mutations of the α regulatory subunit of protein kinase A and up-regulation of the RNA-editing gene transcript in lupus T lymphocytes. Lancet.

[36] Laxminarayana D., O'Rourke K.S., Maas S., Olorenshaw I. (2007). Altered editing in RNA editing adenosine deaminase ADAR2 gene transcripts of systemic lupus erythematosus T lymphocytes. Immunology.

[37] Toyabe S., Kaneko U., Uchiyama M. (2004). Decreased DAP12 expression in natural killer lymphocytes from patients with systemic lupus erythematosus is associated with increased transcript mutations. J. Autoimmun..

[38] Roth S.H., Danan-Gotthold M., Ben-Izhak M., Rechavi G., Cohen C.J., Louzoun Y., Levanon E.Y. (2018). Increased RNA editing may provide a source for autoantigens in systemic lupus erythematosus. Cell Rep..

[39] Shallev L., Kopel E., Feiglin A., Leichner G.S., Avni D., Sidi Y., Eisenberg E., Barzilai A., Levanon E.Y., Greenberger S. (2018). Decreased A-to-I RNA editing as a source of keratinocytes' dsRNA in psoriasis. RNA N. Y. N..

[40] Paz-Yaacov N., Bazak L., Buchumenski I., Porath H.T., Danan-Gotthold M., Knisbacher B.A., Eisenberg E., Levanon E.Y. (2015). Elevated RNA editing activity is a major contributor to transcriptomic diversity in tumors. Cell Rep..

[41] Song C., Sakurai M., Shiromoto Y., Nishikura K. (2016). Functions of the RNA editing enzyme ADAR1 and their relevance to human diseases. Genes.

[42] Loupasakis K., Kuo D., Sokhi U.K., Sohn C., Syracuse B., Giannopoulou E.G., Park S.H., Kang H., Rätsch G., Ivashkiv L.B., Kalliolias G.D. (2017). Tumor Necrosis Factor dynamically regulates the mRNA stabilome in rheumatoid arthritis fibroblast-like synoviocytes. PLoS One.

[43] Hung T., Pratt G.A., Sundararaman B., Townsend M.J., Chaivorapol C., Bhangale T., Graham R.R., Ortmann W., Criswell L.A., Yeo G.W., Behrens T.W. (2015). The Ro60 autoantigen binds endogenous retroelements and regulates inflammatory gene expression. Science.

[44] Heinrich M.J., Purcell C.A., Pruijssers A.J., Zhao Y., Spurlock C.F., Sriram S., Ogden K.M., Dermody T.S., Scholz M.B., Crooke P.S., Karijolich J., Aune T.M. (2019). Endogenous double-stranded Alu RNA elements stimulate IFN-responses in relapsing remitting multiple sclerosis. J. Autoimmun..

[45] Tan M.H., Li Q., Shanmugam R., Piskol R., Kohler J., Young A.N., Liu K.I., Zhang R., Ramaswami G., Ariyoshi K., Gupte A., Keegan L.P., George C.X., Ramu A., Huang N., Pollina E.A., Leeman D.S., Rustighi A., Goh Y.P.S., Chawla A., Del Sal G., Peltz G., Brunet A., Conrad D.F., Samuel C.E., O'Connell M.A., Walkley C.R., Nishikura K., Li J.B., GTEx Consortium, Laboratory (2017). Data analysis &coordinating center (LDACC)—analysis working group, statistical methods groups—analysis working group, enhancing GTEx (eGTEx) groups, NIH common fund, NIH/NCI, NIH/NHGRI, NIH/NIMH, NIH/NIDA, biospecimen collection source site—NDRI, biospecimen collection source site—RPCI, biospecimen core resource—VARI, brain bank repository—university of miami brain endowment bank, leidos biomedical—project management, ELSI study, genome browser data integration &Visualization—EBI, genome browser data integration &Visualization—UCSC genomics institute, university of California santa cruz.

[46] Barturen G., Beretta L., Cervera R., Van Vollenhoven R., Alarcón-Riquelme M.E. (2018). Moving towards a molecular taxonomy of autoimmune rheumatic diseases. Nat. Rev. Rheumatol..

[47] Wang I.X., So E., Devlin J.L., Zhao Y., Wu M., Cheung V.G. (2013). ADAR regulates RNA editing, transcript stability, and gene expression. Cell Rep..

[48] Chung H., Calis J.J.A., Wu X., Sun T., Yu Y., Sarbanes S.L., Dao Thi V.L., Shilvock A.R., Hoffmann H.-H., Rosenberg B.R., Rice C.M. (2018). Human ADAR1 prevents endogenous RNA from triggering translational shutdown. Cell.

[49] Mukherjee N., Corcoran D.L., Nusbaum J.D., Reid D.W., Georgiev S., Hafner M., Ascano M., Tuschl T., Ohler U., Keene J.D. (2011). Integrative regulatory mapping indicates that the RNA-binding protein HuR couples pre-mRNA processing and mRNA stability. Mol. Cell.

[50] Honey K., Rudensky A.Y. (2003). Lysosomal cysteine proteases regulate antigen presentation. Nat. Rev. Immunol..

[51] Liu H., Golji J., Brodeur L.K., Chung F.S., Chen J.T., deBeaumont R.S., Bullock C.P., Jones M.D., Kerr G., Li L., Rakiec D.P., Schlabach M.R., Sovath S., Growney J.D., Pagliarini R.A., Ruddy D.A., MacIsaac K.D., Korn J.M., McDonald E.R. (2019). Tumor-derived IFN triggers chronic pathway agonism and sensitivity to ADAR loss. Nat. Med..

[52] Ishizuka J.J., Manguso R.T., Cheruiyot C.K., Bi K., Panda A., Iracheta-Vellve A., Miller B.C., Du P.P., Yates K.B., Dubrot J., Buchumenski I., Comstock D.E., Brown F.D., Ayer A., Kohnle I.C., Pope H.W., Zimmer M.D., Sen D.R., Lane-Reticker S.K., Robitschek E.J., Griffin G.K., Collins N.B., Long A.H., Doench J.G., Kozono D., Levanon E.Y., Haining W.N. (2019). Loss of ADAR1 in tumours overcomes resistance to immune checkpoint blockade. Nature.

