# Supplementary material for: Increased adenosine-to-inosine RNA editing in rheumatoid arthritis
Source: J Autoimmun. 2020 Jan;106:102329. doi: 10.1016/j.jaut.2019.102329 (PMC7479519; doi:10.1016/j.jaut.2019.102329)
Supplement: Multimedia component 2 [file mmc2.pdf]

Supplementary Material to

Increased adenosine-to-inosine RNA editing in rheumatoid arthritis

Nikolaos I. Vlachogiannis[a,b], Aikaterini Gatsiou[b], Domenico Alessandro Silvestris[c], Kimon Stamatelopoulos[d], Maria G. Tektonidou[a], Angela Gallo[c], Petros P. Sfikakis[a,*], Konstantinos Stellos[b,e,*]

[a]First Department of Propaedeutic Internal Medicine and Joint Rheumatology Program, School of Medicine, National & Kapodistrian University of Athens, Athens, Greece

[b]Cardiovascular Disease Prevention Hub, Faculty of Medical Sciences, Newcastle University, Newcastle upon Tyne, UK

[c]RNA Editing lab, Oncohaematology Dept., Children Hospital Bambino Gesù IRCCS, Rome, Italy

[d]Department of Clinical Therapeutics, Alexandra Hospital, National & Kapodistrian University of Athens, Athens, Greece

[e]Freeman Hospital, Newcastle upon Tyne Hospitals NHS Foundation Trust, Newcastle Upon Tyne, UK

*equal contribution

Supplementary methods

Patient and public involvement

Patients were not included in the design of the current study. However, we plan to disseminate the results to patients' associations and to actively involve patient groups in follow-up studies examining the clinical implications of our results.

Isolation of peripheral blood mononuclear cells

Peripheral blood mononuclear cells (PBMCs) were isolated by ficoll density gradient centrifugation (Ficoll-Paque PLUS, GE Healthcare) within 2 hours from venipuncture, washed twice with phosphate-buffered saline (PBS) containing no Ca/Mg and finally lysed in Trizol (ThermoFisher Scientific) and stored at -80°C, as previously described [1].

RNA isolation and reverse transcription

Total RNA was isolated from patient and control peripheral blood mononuclear cells (PBMCs) using Direct-Zol RNA Miniprep kit (Zymo research) according to manufacturer's instructions with an additional step of DNAse digestion. One(1) μg of total RNA was reverse transcribed into cDNA using the MuLV reverse transcriptase kit (Invitrogen), as previously described [1].

Quantitative polymerase chain reaction

For the quantification of the two ADAR1 isoforms (ADAR1p110 and ADAR1p150) specifically designed Taqman primers were used (ADAR1p110: Hs01017596; ADAR1p150: Hs01020780, Applied Biosystems) while TATA-Box binding protein (TBP) (Hs00427621, Applied Biosystems) served as the housekeeping gene. Quantification of cathepsin S and human antigen R (HuR) was conducted with Takara SYBR Premix Ex Taq, while RPLP0 served as housekeeping gene. The relative expression of each gene was determined according to the formula $2^{-\Delta Ct}$ ($\Delta Ct = Ct_{(gene)} - Ct_{(housekeeping\ gene)}$).

Sequences of primers used for qPCR:

RPLP0: F:5'-TCGACAATGGCAGCATCTAC-3',

R:5'-ATCCGTCTCCACAGACAAGG-3';

cathepsin S *(CTSS):* F:5'-TCATACGATCTGGGCATGAA-3',

R:5'-AGGTTCTGGGCACTGAGAGA-3';

HuR (*ELAVL1*) F:5'-GAAGACCACATGGCCGAAGA-3',

R:5'-CCAAGCTGTGTCCTGCTACT-3'[1].

Sample size calculation

In terms of power considerations, a total sample size of 20 subjects allocated in two equal groups would provide adequate power (i.e. over 80%) to detect a difference of 0.45 units of relative ADAR1p150 expression levels by the non-parametric Mann-Whitney test for independent samples. Measures of dispersion and anticipated differences were derived from pilot data from an interim analysis of our study (5 cases and 5 controls, mean± SD 2.104±0.363 versus 1.637±0.283, respectively). A priori power analysis was performed with G*Power v 3.1.9.4 [2].

Supplementary figures and tables

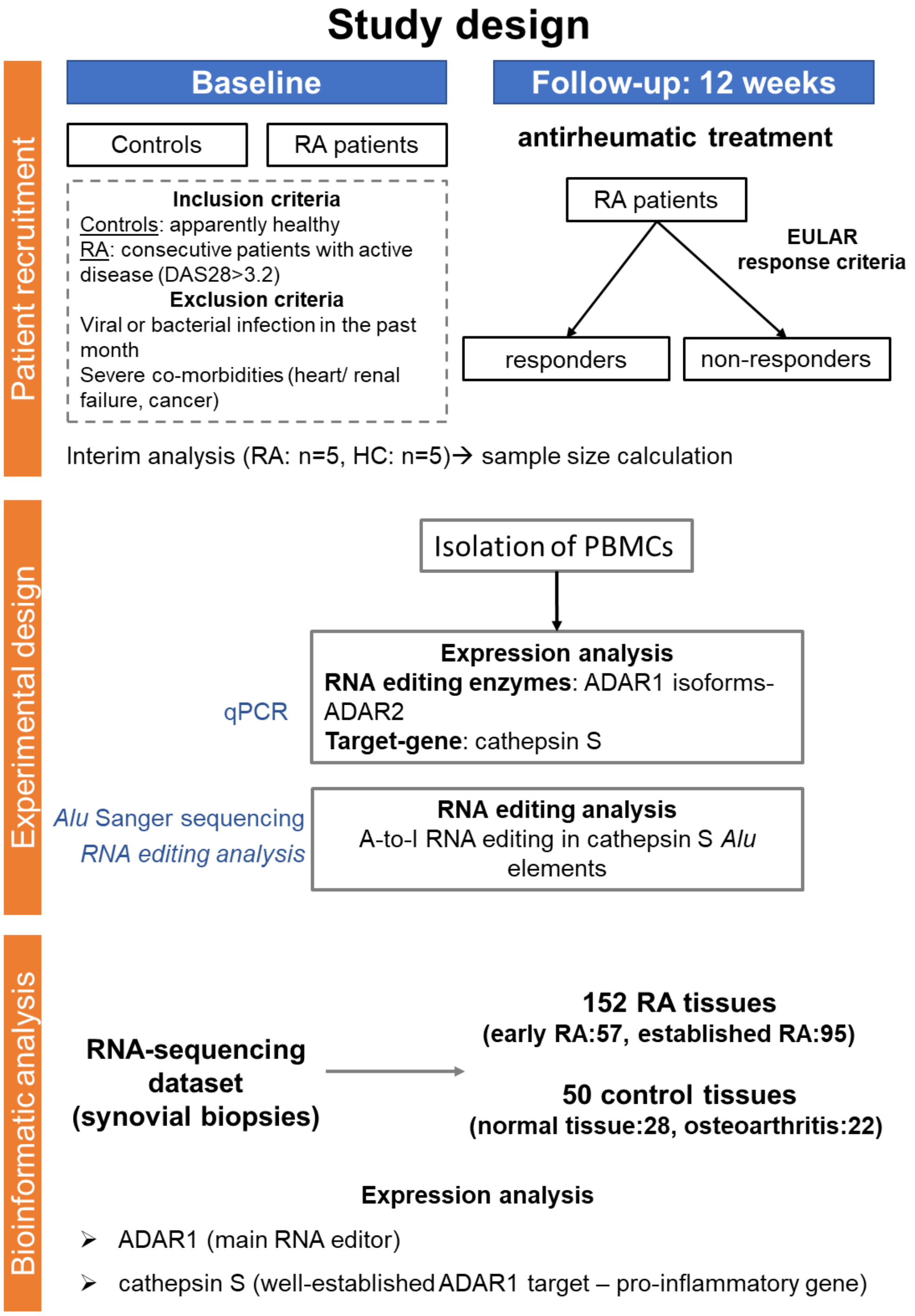

Supplementary figure 1. Schematic representation of study design

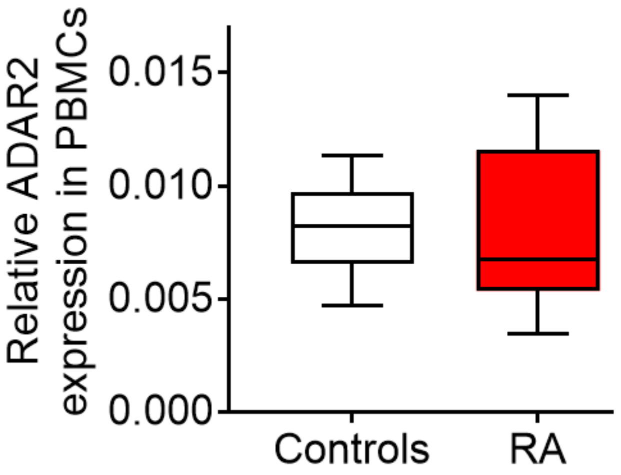

Supplementary figure 2: mRNA expression of the second RNA editor, ADAR2 (*ADARB1*), in peripheral blood mononuclear cells of patients with RA (n=19) and healthy controls (n=14).

Supplementary Table 1. Correlation of RNA editing rate of individual adenosines located in cathepsin S 3' UTR *AluSx*$^{+}$ with ADAR1p150/ ADAR1p110 and cathepsin S mRNA expression in RA PBMCs

	ADAR1p150	ADAR1p110	Cathepsin S
A1662	**r=0.598 (P=0.007)**	**r=0.546 (P=0.02)**	r=0.249 (P=0.30)
A1672	**r=0.463 (P<0.05)**	**r=0.512 (P=0.03)**	r=0.446 (P=0.06)
A1684	**r=0.740 (P<0.001)**	r=0.405 (P=0.09)	**r=0.498 (P=0.03)**
A1710	**r=0.611 (P=0.005)**	r=0.325 (P=0.18)	r=0.318 (P=0.19)
A1735	**r=0.619 (P=0.005)**	r=0.423 (P=0.07)	r=0.449 (P>0.05)
A1758	**r=0.707 (P=0.001)**	**r=0.518 (P=0.02)**	**r=0.472 (P=0.04)**
A1770	**r=0.700 (P=0.001)**	**r=0.495 (P=0.03)**	**r=0.558 (P=0.01)**
A1777	r=0.202 (P=0.41)	r=-0.08 (P=0.74)	r=0.089 (P=0.72)
A1780	**r=0.616 (P=0.005)**	r=0.386 (P=0.10)	r=0.400 (P=0.09)
A1802	**r=0.521 (P=0.02)**	r=0.325 (P=0.18)	**r=0.537 (P=0.02)**
A1806	**r=0.670 (P=0.002)**	r=0.393 (P=0.10)	**r=0.632 (P=0.004)**
A1807	**r=0.595 (P=0.007)**	r=0.344 (P=0.15)	**r=0.540 (P=0.02)**
A1815	**r=0.647 (P=0.003)**	r=0.388 (P=0.10)	r=0.437 (P=0.06)
A1821	**r=0.754 (P<0.001)**	**r=0.493 (P=0.03)**	**r=0.581 (P=0.009)**
A1824	**r=0.454 (P=0.05)**	r=0.263 (P=0.28)	**r=0.716 (P=0.001)**
A1825	r=0.440 (P=0.06)	r=0.254 (P=0.29)	**r=0.533 (P=0.02)**
A1826	**r=0.728 (P<0.001)**	r=0.446 (P=0.06)	**r=0.558 (P=0.01)**
A1830	r=0.388 (P=0.10)	r=0.188 (P=0.44)	r=0.284 (P=0.24)
A1843	r=0.314 (P=0.19)	r=0.005 (P=0.98)	r=0.209 (P=0.39)
A1846	r=0.435 (P=0.06)	r=0.186 (P=0.45)	**r=0.461 (P<0.05)**
A1850	r=0.328 (P=0.17)	r=0.018 (P=0.94)	r=0.346 (P=0.15)
A1888	r=-0.019 (P=0.94)	r=-0.188 (P=0.44)	r=0.000 (P=1.00)
A1903	r=0.140 (P=0.57)	r=0.132 (P=0.59)	r=0.260 (P=0.28)
A1909	**r=0.616 (P=0.005)**	**r=0.465 (P<0.05)**	**r=0.628 (P=0.004)**

A signifies the nucleotide position of each adenosine in cathepsin S 3' UTR. Correlations were examined with Spearman’s rank test. Statistical significance was set at P≤0.05

Supplementary Table 2. *Alu*-enriched molecules involved in TNF-signaling pathway predicted to be edited

	Number of *Alu* elements	Number of predicted editing sites in *Alus*	Correlation with ADAR1 in RNA-seq. (GSE89408, n=152)
**TRAF1**	10	18	**r=0.508 (P<0.001)**
**TRAF2**	45	292	**r=0.732 (P<0.001)**
**TRAF3**	117	1135	**r=0.743 (P<0.001)**
**TRAF5**	24	123	**r=0.356 (P<0.001)**
**RIPK1**	24	119	**r=0.637 (P<0.001)**

Number of *Alu* elements was extracted from transpogene database (http://transpogene.tau.ac.il), while predicted A-to-I RNA editing sites were extracted from RADAR RNA editing database (http://rnaedit.com). Correlation of Alu-enriched genes with ADAR1 was examined in the RA samples of RNA-seq. dataset GSE89408 using Spearman's rank test.

References

[1] K. Stellos, A. Gatsiou, K. Stamatelopoulos, L. Perisic Matic, D. John, F.F. Lunella, N. Jaé, O. Rossbach, C. Amrhein, F. Sigala, R.A. Boon, B. Fürtig, Y. Manavski, X. You, S. Uchida, T. Keller, J.-N. Boeckel, A. Franco-Cereceda, L. Maegdefessel, W. Chen, H. Schwalbe, A. Bindereif, P. Eriksson, U. Hedin, A.M. Zeiher, S. Dimmeler, Adenosine-to-inosine RNA editing controls cathepsin S expression in atherosclerosis by enabling HuR-mediated post-transcriptional regulation, Nat. Med. 22 (2016) 1140–1150. doi:10.1038/nm.4172.

[2] F. Faul, E. Erdfelder, A.-G. Lang, A. Buchner, G*Power 3: A flexible statistical power analysis program for the social, behavioral, and biomedical sciences, Behav. Res. Methods. 39 (2007) 175–191. doi:10.3758/BF03193146.
